# A simple method to resolve rate constants when the binding mechanism obeys induced fit or conformational selection

**DOI:** 10.1016/j.jbc.2024.107131

**Published:** 2024-03-02

**Authors:** Enrico Di Cera

**Affiliations:** Edward A. Doisy Department of Biochemistry and Molecular Biology, Saint Louis University School of Medicine, St. Louis, Missouri, USA

**Keywords:** presteady-state kinetics, enzyme mechanism, serine protease, thrombin, prothrombin

## Abstract

Many interactions involving a ligand and its molecular target are studied by rapid kinetics using a stopped-flow apparatus. Information obtained from these studies is often limited to a single, saturable relaxation that is insufficient to resolve all independent rate constants even for a two-step mechanism of binding obeying induced fit (IF) or conformational selection (CS). We introduce a simple method of general applicability where this limitation is overcome. The method accurately reproduces the rate constants for ligand binding to the serine protease thrombin determined independently from the analysis of multiple relaxations. Application to the inactive zymogen precursor of thrombin, prethrombin-2, resolves all rate constants for a binding mechanism of IF or CS from a single, saturable relaxation. Comparison with thrombin shows that the prethrombin-2 to thrombin conversion enhances ligand binding to the active site not by improving accessibility through the value of *k*_on_ but by reducing the rate of dissociation *k*_off_. The conclusion holds regardless of whether binding is interpreted in terms of IF or CS and has general relevance for the mechanism of zymogen activation of serine proteases. The method also provides a simple test of the validity of IF and CS and indicates when more complex mechanisms of binding should be considered.

Several systems in biology are studied in terms of rapid kinetics to arrive at the mechanism that governs ligand binding to its macromolecular target (1). The simplest interpretation envisions a rigid body docking of the ligand onto its target and is known as the lock-and-key mechanism (2). In this case, investigation of the system by rapid kinetics yields a rate of relaxation to equilibrium that increases linearly with the ligand concentration (1, 3, 4, 5). More often, however, the binding step is linked to conformational transitions that follow or precede the binding step. The former case defines the induced fit (IF) mechanism first proposed by Koshland (6). The latter case defines the pre-equilibrium mechanism first proposed by Eigen (7), also known as conformational selection (CS) (8). The presence of a conformational transition linked to binding manifests itself with an additional relaxation that saturates as a function of ligand concentration to indicate interconversion between two states. The relevant kinetic schemes for IF and CS are

(1) $$E\begin{array}{c} k_{on}x\\ ⇄\\ k_{off} \end{array}EX\begin{array}{c} k_{23}\\ ⇄\\ k_{32} \end{array}E^{\prime }X$$

(2) $$E^{*}\begin{array}{c} k_{12}\\ ⇄\\ k_{21} \end{array}E\begin{array}{c} k_{on}x\\ ⇄\\ k_{off} \end{array}EX$$

where E denotes the macromolecule or biological target binding ligand X, whose concentration is x. The binding step in the two schemes corresponds to the rigid body interaction of the lock-and-key mechanism. In the IF mechanism (Equation 1), the binding step is followed by a conformational transition of the complex from EX to $E^{\prime }X$. In the CS mechanism (Equation 2), the macromolecule preexists in two conformations, *E∗* and *E*, and binding takes place exclusively to the E conformation. Both schemes of IF and CS contain four independent rate constants that must be resolved from analysis of experimental data. The rate constant $k_{on}$ has dimensions of M^−1^s^−1^ and defines the second order rate of diffusion of the ligand into the binding site. This is a property of the free species and depends on physical, chemical or structural variables that affect the ligand and/or the macromolecule before the complex is formed (1). The rate constant $k_{off}$ has dimensions of s^−1^ and defines the first-order rate of dissociation of the complex EX into the parent species E and X. As such, the constant reflects properties of the complex and is influenced by physical, chemical, or structural variables that affect such complex. The rate constants $k_{12}$, $k_{21}$, $k_{23}$, and $k_{32}$ are also first-order and define the rates of transition between alternative conformations of the macromolecule in the free ($k_{12}$, $k_{21}$ for CS) or bound ($k_{23}$, $k_{32}$ for IF) forms. The values of $k_{on}$ and $k_{off}$ define the intrinsic dissociation constant at equilibrium, $K_{d}=k_{off}/k_{on}$ (9), which measures the strength of interaction and the relative affinity of different ligands binding to the same target or of the same ligand binding to different targets. Changes in $K_{d}$ also give information on how the interaction is affected by changes in solution conditions, temperature, pressure, or mutations introduced in the system (9).

In general, a kinetic mechanism generates a number of linear and saturable relaxations equal to the number of independent binding and conformational transitions involved (1, 10). There are three species in the reaction schemes for IF and CS. Because the system is closed and mass is conserved, only two of them are independent and give rise to two independent relaxations. The former reflects binding and increases linearly with the ligand concentration. The latter reflects the linked conformational transition and saturates at high ligand concentrations. These are the properties that can be accessed experimentally by rapid kinetics using a stopped-flow apparatus when studying how the interaction reaches equilibrium after the initial mixing of ligand and its target (11, 12, 13). However, a common situation encountered in practice presents the experimentalist only with a single, saturable relaxation that documents the presence of a conformational transition linked to binding but the binding step is itself too fast to measure (1, 3, 4, 5, 14). This complicates resolution of the independent rate constants and a full characterization of the mechanism of binding. Here, we show how this difficulty can be overcome with the use of a simple method that applies generally to IF and CS and also serves as a test of the validity of these mechanisms.

## Results

### Mathematical equivalence of IF and CS

**Figure 1:**
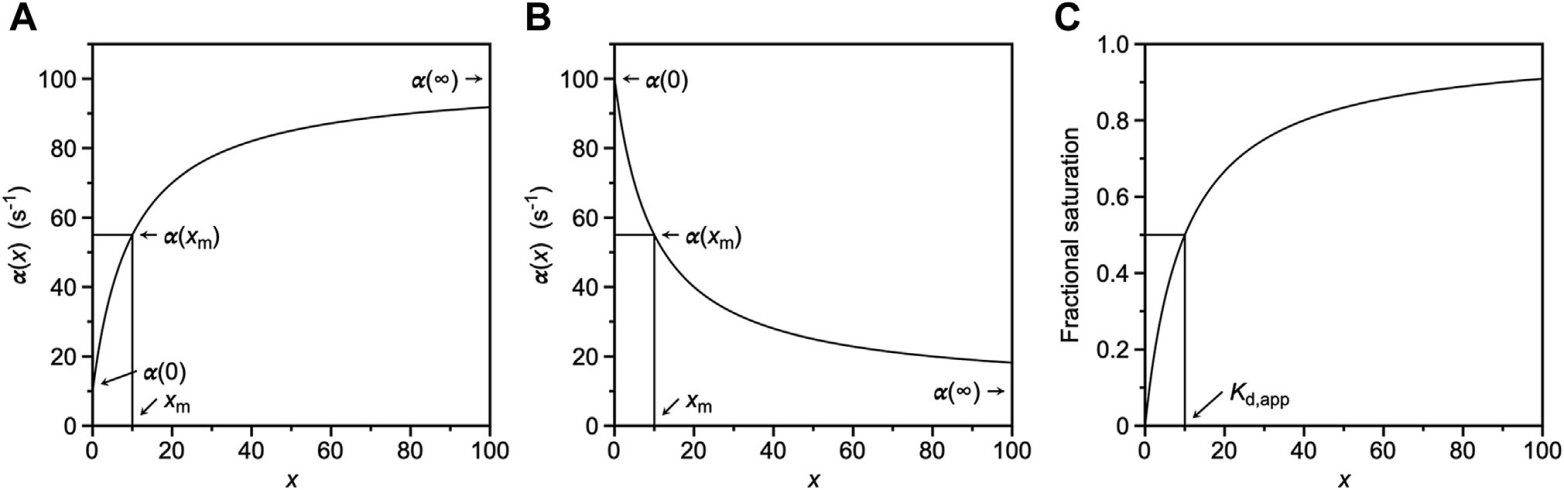
**Relevant plots needed for the derivation of the value of**$k_{on}$**from**Equation 11**using the method discussed in the text.***A*, dependence of the slow relaxation, α(x), increasing with the ligand concentration x for a binding mechanism obeying IF (Equation 1) or CS (Equation 2). Shown are the model-independent parameters α(0), α(∞), $\alpha \left(x_{m}\right)$, and $x_{m}$ that enable derivation of the value of $k_{on}$ with the additional information obtained from equilibrium data (see panel *C*). The parameters can also be used to extract information on the rate constants of the IF and CS mechanisms (Table 2) by virtue of their mathematical equivalence illustrated by the conversion formulas in Table 1. *B*, dependence of the slow relaxation, α(x), decreasing with the ligand concentration x for a binding mechanism obeying CS (Equation 2). The dependence is incompatible with IF. Shown are the parameters α(0), α(∞), $\alpha \left(x_{m}\right)$, and $x_{m}$ (see also panel *A*). *C*, fractional saturation $x/\left(K_{d,app}+x\right)$ plotted as a function of the ligand concentration x obtained by equilibrium titrations (see Equation 13). The midpoint of the curve defines the value of $K_{d,app}$ that, together with the parameters obtained from the slow relaxation (*A* and *B*), yields the value of $k_{on}$ from Equation 11. CS, conformational selection; IF, induced fit.

The properties of IF (Equation 1) and CS (Equation 2) have been discussed elsewhere (1, 3, 4, 5, 15). The two mechanisms are mathematically irreducible and easily distinguished from analysis of experimental data under the so-called rapid equilibrium approximation, that is, when the binding step takes place on a time scale considerably faster than the linked conformational transition. The kinetic expressions simplify under such approximation: the saturable relaxation associated with the conformational transition increases with the ligand concentration x when the mechanism obeys IF and decreases with x when CS applies (1, 5). Because the former behavior predominates in systems of interest to biochemistry and pharmacology, it has been concluded that IF is a dominant mechanism of recognition in biology (5, 16). However, when the simplification introduced by the rapid equilibrium approximation is dropped and the kinetic expressions for IF and CS are derived in their general form, a different conclusion is arrived at (11). When the saturable relaxation increases with the ligand concentration x (Fig. 1*A*), IF applies but CS cannot be ruled out. Distinguishing between the two mechanisms becomes necessary in this case, as discussed in detail elsewhere (1, 13, 17, 18, 19, 20). On the other hand, when the saturable relaxation decreases (Fig. 1*B*), only CS applies and IF is ruled out. Therefore, CS is more general than IF as a mechanism of ligand binding because, unlike IF, it can never be ruled out *a priori* from inspection of the saturable relaxation. More importantly, IF is a mathematical special case of CS (21), which implies that any experimental dataset compatible with IF (Equation 1) can be interpreted with identical accuracy in terms of CS (Equation 2), but the reverse is not true. The conversion formulas that underscore this equivalence are summarized in Table 1. Our discussion will therefore deal with the mathematical expressions of CS, without loss of generality.

**Table 1:** Conversion formulas between IF and CS

From IF to CS
$k_{on}^{CS}=k_{on}^{IF}$
$2k_{off}^{CS}=k_{off}^{IF}+k_{23}+k_{32}-\sqrt{{\left(k_{off}^{IF}-k_{23}-k_{32}\right)}^{2}+4{k_{off}^{IF}k}_{23}}$
$k_{12}=k_{23}+k_{32}$
$2k_{21}=k_{off}^{IF}-k_{23}-k_{32}+\sqrt{{\left(k_{off}^{IF}-k_{23}-k_{32}\right)}^{2}+4{k_{off}^{IF}k}_{23}}$
From CS to IF
$k_{on}^{IF}=k_{on}^{CS}$
$k_{off}^{IF}=k_{off}^{CS}+k_{21}$
$k_{23}=k_{21}{\left(k_{12}-k_{off}^{CS}\right)\left({k_{21}+k}_{off}^{CS}\right)}^{-1}$
$k_{32}=k_{off}^{CS}\left(k_{12}+k_{21}\right){\left({k_{21}+k}_{off}^{CS}\right)}^{-1}$

Rate constants refer to Equations 1 and 2, with superscript denoting the values for IF and CS.

Abbreviations: CS, conformational selection; IF, induced fit.

### Resolving all rate constants of the CS scheme when only the saturable relaxation is available

Under conditions most commonly encountered in practice, data are collected with the ligand in large excess over the macromolecule and the two independent relaxations associated with CS in Equation 2 are (1)

(3) $${2\alpha }_{1}\left(x\right)=k_{on}x+k_{off}+k_{12}+k_{21}+\sqrt{{\left(k_{on}x+k_{off}-k_{12}-k_{21}\right)}^{2}+4k_{21}k_{on}x}$$

(4) $${2\alpha }_{2}\left(x\right)=k_{on}x+k_{off}+k_{12}+k_{21}-\sqrt{{\left(k_{on}x+k_{off}-k_{12}-k_{21}\right)}^{2}+4k_{21}k_{on}x}$$

We are interested in resolving the four independent rate constants in Equation 2 when only the saturable relaxation $\alpha_{2}\left(x\right)$ can be measured experimentally, which is a common outcome in rapid kinetics studies (1, 3, 4, 5, 14). We will henceforth refer to this relaxation as α(x) for simplicity and focus our treatment on the rate constant $k_{on}$, which can only be derived from the fast relaxation (Equation 3) as the slope in the limit of large x (1). We also note from the conversion formulas in Table 1 that the value of $k_{on}$ is the same for IF and CS, which makes determination of this rate constant independent of the reaction scheme used in the analysis of experimental data. The mathematical properties of Equation 4 are discussed elsewhere (1) and enable resolution of three of the four independent rate constants from the following three constraints, that is, the asymptotic values

(5) $$\alpha \left(0\right)={smaller\;of\;k}_{off}\;or\;k_{12}+k_{21}$$

(6) $$\alpha \left(\infty \right)=k_{12}$$

and the midpoint of the transition, $x_{m}$, where

(7) $$2\alpha \left(x_{m}\right)={\alpha \left(0\right)+\alpha \left(\infty \right)=k}_{on}x_{m}+k_{off}+k_{12}+k_{21}-\sqrt{{\left(k_{on}x_{m}+k_{off}-k_{12}-k_{21}\right)}^{2}+4k_{21}k_{on}x_{m}}$$

These values can be derived from inspection of the slow relaxation, whether it increases (Fig. 1*A*) or decreases (Fig. 1*B*) with x. When the slow relaxation increases, the parameters in Equations (5), (6), (7) can also be interpreted in terms of IF using the conversion formulas in Table 1.

Assignment of the lower asymptote α(0) requires knowledge of the relative values of $k_{off}$ and $k_{12}+k_{21}$. The relaxation decreases with x when α(0)= $k_{12}+k_{21}$ or $k_{off}>k_{12}$, increases with x when $k_{off}<k_{12}$ and is independent of x when $k_{off}=k_{12}$ (1, 11). However, regardless of the value of α(0), Equation 7 can be expanded and rearranged into

(8) $$k_{on}x_{m}\left[\alpha \left(x_{m}\right)-k_{12}\right]=\left[\alpha \left(x_{m}\right)-k_{off}\right]\left[\alpha \left(x_{m}\right)-k_{12}-k_{21}\right]$$

The expression is symmetric in $k_{off}$ and $k_{12}+k_{21}$, that is, it assumes the same value regardless of whether α(0) is defined as the smaller of $k_{off}$ or $k_{12}+k_{21}$. The other term, that is, the larger of $k_{off}$ or $k_{12}+k_{21}$ can be used to define $\hat{\alpha }\left(0\right)$. Hence, by virtue of Equations 6 and 7,

(9) $$k_{on}x_{m}=\frac{\left[\alpha \left(x_{m}\right)-k_{off}\right]\left[\alpha \left(x_{m}\right)-k_{12}-k_{21}\right]}{\left[\alpha \left(x_{m}\right)-k_{12}\right]}=\frac{\left[\alpha \left(x_{m}\right)-\alpha \left(0\right)\right]\left[\alpha \left(x_{m}\right)-\hat{\alpha }\left(0\right)\right]}{\left[\alpha \left(x_{m}\right)-\alpha \left(\infty \right)\right]}=\hat{\alpha }\left(0\right)-\alpha \left(x_{m}\right)$$

The expression in Equation 9 shows that resolution of $k_{on}$ requires knowledge of $\hat{\alpha }\left(0\right)$, which cannot be derived by inspection or analysis of the slow relaxation. Additional information is needed and can be obtained from the apparent equilibrium dissociation constant for the CS mechanism in Equation 2 (1), that is,

(10) $$K_{d,app}=K_{d}\frac{k_{12}+k_{21}}{k_{12}}=\frac{k_{off}}{k_{on}}\frac{k_{12}+k_{21}}{k_{12}}=\frac{1}{k_{on}}\frac{\alpha \left(0\right)\hat{\alpha }\left(0\right)}{\alpha \left(\infty \right)}$$

The expression in Equation 10 is also symmetric in $k_{off}$ and $k_{12}+k_{21}$ or α(0) and $\hat{\alpha }\left(0\right)$. Therefore, eliminating the unknown quantity $\hat{\alpha }\left(0\right)$ between Equations 9 and 10 yields

(11) $$k_{on}=\frac{\alpha \left(x_{m}\right)}{K_{d,app}\frac{\alpha \left(\infty \right)}{\alpha \left(0\right)}-x_{m}}$$

which is independent of how α(0) is defined and whether α(x) increases or decreases with x. The mathematical equivalence between IF and CS (Table 1) makes the value of $k_{on}$ identical in the two mechanisms (21) and gives Equation 11 general applicability in practical applications. Remarkably, all the terms in Equation 11 can be derived from inspection of the slow relaxation (Fig. 1, *A* and *B*) and the equilibrium binding curve (Fig. 1*C*), without the need for data analysis using the expressions for IF or CS. The value of $k_{on}$ as an important property of the binding mechanism can be extracted directly from experimental data through application of Equation 11, regardless of whether the mechanism obeys IF or CS.

### Application of the method

Along with Equations (5), (6), (7), Equation 11 provides a fourth constraint needed for the resolution of all four independent rate constants defining IF (Equation 1) or CS (Equation 2). This has important implications for practical applications. Prethrombin-2 is the direct zymogen precursor of the coagulation protease thrombin and an intermediate along the pathway of prothrombin activation (22, 23). As for many serine proteases of the trypsin family (24, 25, 26), the conversion of prethrombin-2 to thrombin obeys the celebrated Huber-Bode mechanism of zymogen activation (27). In this mechanism, the zymogen is cleaved at a conserved Arg residue in the activation domain and the new N terminus penetrates the active site, where a new H-bond with the highly conserved residue D194 organizes the catalytic triad and primary specificity pocket to yield the mature protease (27). The zymogen to protease conversion enables efficient catalysis of substrate and enhances binding to the active site. For example, the active site inhibitor argatroban binds to the mature protease thrombin with an affinity 200-fold higher than to the zymogen prethrombin-2 in an enthalpically driven interaction promoted by formation of specific H-bonds (28). However, the kinetic components of this enhanced affinity remain undefined for this and other zymogen to protease transitions in the trypsin family because of the difficulty of measuring the underlying rate constants, especially for the zymogen form (24, 25, 26, 29). Specifically, it is unclear whether the significant enhancement in binding affinity observed for the protease is due to increased $k_{on}$, decreased $k_{off}$, or both. The additional H-bonds documented in the crystal structure of the thrombin–argatroban complex relative to the prethrombin-2–argatroban complex (22) may promote a higher $k_{on}$ but also anchor the inhibitor more tightly and decrease $k_{off}$.

**Figure 2:**
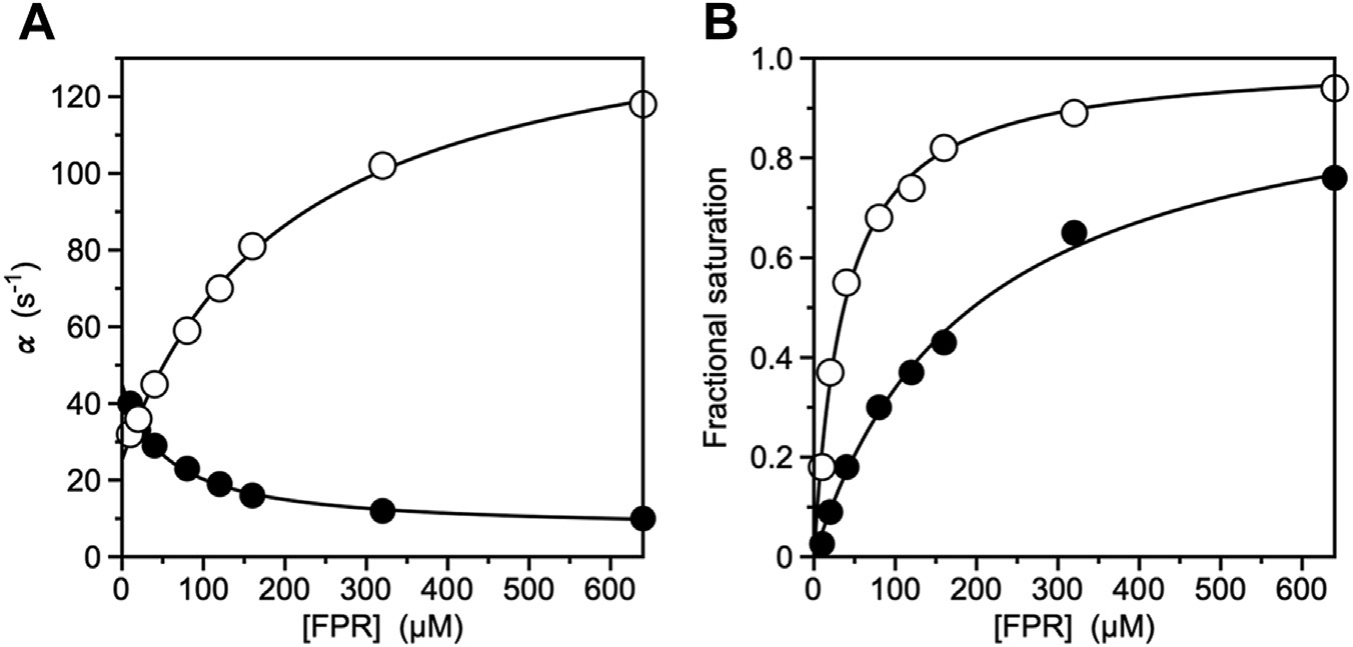
**Binding of FPR to thrombin zymogens.***A*, rapid kinetics of FPR binding to the zymogens prothrombin (*closed circles*) and prethrombin-2 (*open circles*), showing a saturable relaxation associated with the interaction. The lack of information on a fast relaxation increasing linearly with [FPR] prevented assignment of the value of $k_{on}$ in the original report (30). Curves were drawn with the empirical expression $\left[\alpha \left(0\right)x_{m}+\alpha \left(\infty \right)x\right]/\left(x_{m}+x\right)$, where x =[FPR], with best-fit parameters listed in Table 2. *B*, fractional saturation of FPR binding to prothrombin (*closed circles*) and prethrombin-2 (*open circles*). Curves were drawn with the expression $x/\left(K_{d,app}+x\right)$, where *x* = [FPR], with best-fit values of $K_{d,app}$ listed in Table 2. These values, together with the parameters derived from the plots in panel *A*, allow for derivation of the value of $k_{on}$ from Equation 11. The value for prethrombin-2 (4.0 ± 0.4 μM^−1^s^−1^) is comparable to that obtained for the mature protease thrombin (Table 2). On the other hand, the value derived for prothrombin is negative and shows that the mechanism for FPR binding to this zymogen does not obey IF or CS and must involve additional kinetic steps. Data in *panels A and B* were taken from (30) and were obtained under the following experimental conditions: 400 mM ChCl, 50 mM Tris, 0.1% PEG8000, pH 8, at 15 °C. CS, conformational selection; FPR, H-D-Phe-Pro-Arg-p-nitroanilide; IF, induced fit.

Recent measurements of the binding of the chromogenic substrate H-D-Phe-Pro-Arg-p-nitroanilide (FPR) to the S195A mutants of thrombin and prethrombin-2, inactivated at the catalytic S195 to prevent hydrolysis, have reported values of $K_{d,app}$ = 0.28 ± 0.02 μM and 37 ± 4 μM for the two proteins (30), consistent with the results on argatroban binding (28). Rapid kinetics of FPR binding to thrombin resolved the two independent relaxations (Equations 3 and 4) for CS and the four independent rate constants. On the other hand, similar measurements for prethrombin-2 produced only a saturable relaxation increasing with [FPR] (Fig. 2*A*) (30), thereby preventing resolution of the four independent constants and a full comparison with the mature protease thrombin. This situation provides an ideal test for application of the method based on Equation 11. The saturable relaxation for FPR binding to prethrombin-2 increases from α(0) = 25 ± 0.3 s^−1^ to α(∞) = 150 ± 20 s^−1^, with a midpoint $x_{m}$ = 200 ± 20 μM and $\alpha \left(x_{m}\right)$ = 88 ± 9 s^−1^ (Fig. 2*A* and Table 2). A value of $K_{d,app}$ = 37 ± 4 μM for FPR binding at equilibrium was obtained by fluorescence titrations under identical solution conditions (Fig. 2*B*) (30). Interpretation of the saturable relaxation in terms of CS gives $k_{12}$ = 150 ± 20 s^−1^ and $k_{off}$ = 25 ± 3 s^−1^. Application of Equation 11 then yields $k_{on}$ = 4.0 ± 0.4 μM^−1^s^−1^ and Equation 9 gives $\hat{\alpha }\left(0\right)={k_{12}+k}_{21}$ = 890 ± 90 s^−1^, so $k_{21}$ = 740 ± 70 s^−1^ (Table 2). The value of $k_{on}$ is almost identical to that reported for the mature protease thrombin (2.6 ± 0.3 μM^−1^s^−1^), whilst the value of $k_{off}$ is 40-fold higher (25 ± 3 s^−1^ *versus* 0.60 ± 0.06 s^−1^) (30). Hence, the higher binding affinity in the protease is not due to enhanced rate of diffusion into the active site but to decreased rate of dissociation of the complex into the parent species. The conclusion is independent of whether FPR binding to prethrombin-2 is interpreted according to CS or IF. Application of the conversion formulas in Table 1 gives rate constants for IF equal to $k_{on}$ = 4.0 ± 0.4 μM^−1^s^−1^, $k_{off}$ = 765 ± 80 s^−1^, $k_{23}$ = 120 ± 10 s^−1^, and $k_{32}$ = 29 ± 3 s^−1^ (Table 2), thereby making the contribution of a decreased rate of dissociation in the protease even more evident. Importantly, the two sets of rate constants for IF and CS yield the same value for $K_{d,app}$ (Table 2), as shown by application of Equation 10 and the conversion formulas in Table 1. The importance of $k_{off}$ in setting the difference between ligand binding to prethrombin-2 and thrombin was anticipated by previous studies (30). The method based on Equation 11 provides a quantitative estimate of the difference and adds rigor to previous conclusions.

**Table 2:** Parameters for FPR binding

	Thrombin	Prethrombin-2	Prothrombin
Model independent			
α(0) (s^−1^)	0.60 ± 0.06	25 ± 2	45 ± 5
α(∞) (s^−1^)	17 ± 2	150 ± 10	6.9 ± 0.7
$\alpha \left(x_{m}\right)$ (s^−1^)	8.8 ± 0.9	88 ± 9	26 ± 3
$x_{m}$ (μM)	4.3 ± 0.4	200 ± 20	54 ± 5
$K_{d,app}$ (μM)	0.28 ± 0.02	37 ± 4	195 ± 20
$k_{on}$ (μM^−1^s^−1^)^a^	2.4 ± 0.2	4.0 ± 0.4	−1.1
CS			
$k_{on}^{CS}$ (μM^−1^s^−1^)	2.4 ± 0.2 (2.6 ± 0.3)	4.0 ± 0.4	n/a
$k_{off}^{CS}$ (s^−1^)	0.60 ± 0.06 (0.6 ± 0.1)	25 ± 3	n/a
$k_{12}$ (s^−1^)	17 ± 2 (16 ± 1)	150 ± 10	n/a
$k_{21}$ (s^−1^)	2.0 ± 0.2 (3.8 ± 0.8)	740 ± 70	n/a
IF			
$k_{on}^{IF}$ (μM^−1^s^−1^)	2.4 ± 0.2 (2.6 ± 0.3)	4.0 ± 0.4	n/a
$k_{off}^{IF}$ (s^−1^)	2.6 ± 0.3 (4.4 ± 0.4)	765 ± 80	n/a
$k_{23}$ (s^−1^)	13 ± 1 (13 ± 1)	120 ± 10	n/a
$k_{32}$ (s^−1^)	4.4 ± 0.4 (2.6 ± 0.3)	29 ± 3	n/a

Abbreviations: CS, conformational selection; FPR, H-D-Phe-Pro-Arg-p-nitroanilide; IF, induced fit.

a Derived from application of Equation 11 in the text. Values refer to application of the method discussed in the text. Values in parentheses are from (30).

### Validation of the method

**Figure 3:**
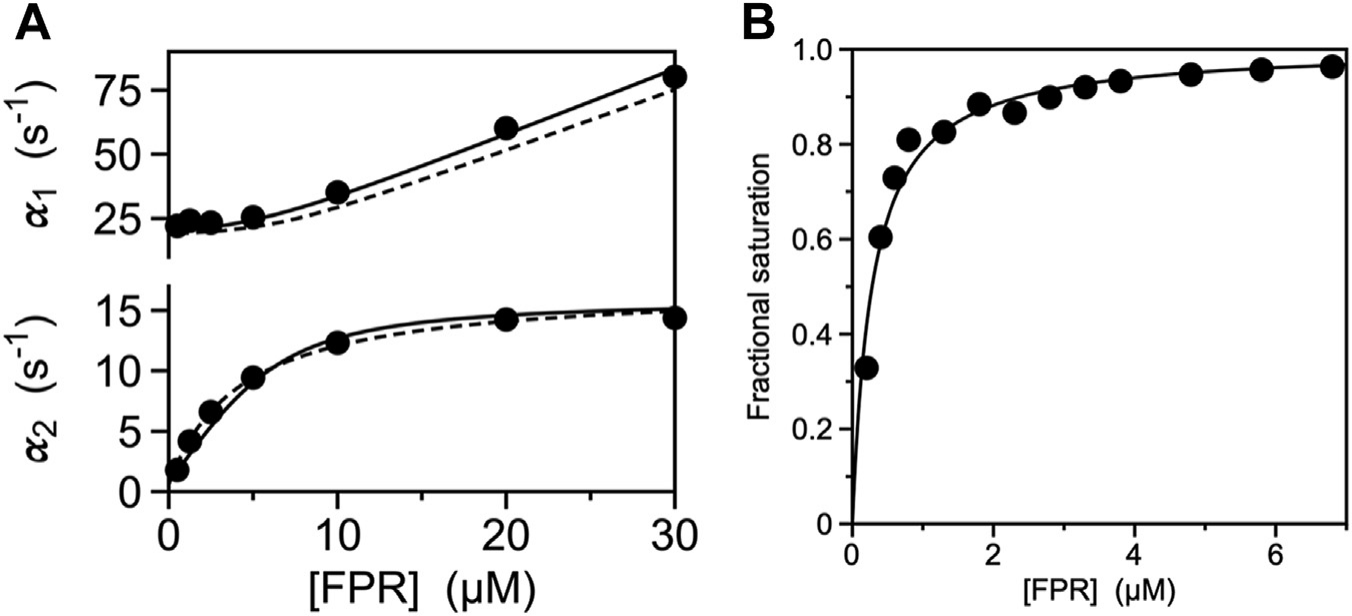
**Binding of FPR to thrombin.***A*, rapid kinetics of FPR binding to thrombin showing the fast ($\alpha_{1}$) and slow ($\alpha_{2}$) relaxations defining a mechanism of binding consistent with IF or CS (see also Fig. 1*A*). Continuous lines depict Equations 3 and 4 in the original report (30) with best-fit parameter values of Equation 1 or Equation 2 listed in Table 2. The method presented in the text was used to analyze these data by assuming no knowledge of the fast relaxation. The discontinuous line for the slow relaxation was drawn with the empirical expression $\left[\alpha \left(0\right)x_{m}+\alpha \left(\infty \right)x\right]/\left(x_{m}+x\right)$, where x = [FPR], with best-fit parameters listed in Table 2. The discontinuous line for the fast relaxation was drawn using Equation 3 with the four independent rate constants obtained from application of Equation 11 (Table 2). The predicted fast relaxation (*discontinuous line*) approximates quite well the actual experimental data and lends confidence to the validity of the method. *B*, fractional saturation of FPR binding to thrombin. The curve was drawn with the expression $x/\left(K_{d,app}+x\right)$, where x = [FPR], with a best-fit value of $K_{d,app}$ = 0.28 ± 0.02 μM (30). This value, together with the parameters derived from analysis of the slow relaxation in panel *A*, allows for derivation of the value of $k_{on}$ from Equation 11. The four independent rate constants derived from application of the method compare well with the values determined from application of Equations 3 and 4 in the original report (30). Data in panels *A* and *B* were taken from (30) and were obtained under the following experimental conditions: 400 mM ChCl, 50 mM Tris, 0.1% PEG8000, pH 8, at 15 °C. CS, conformational selection; FPR, H-D-Phe-Pro-Arg-p-nitroanilide; IF, induced fit.

An important validation of the method is offered by application to a case where all rate constants have been determined from analysis of the two relaxations (Equations 3 and 4). In this case, one can assume no knowledge of the fast relaxation and consider only the slow relaxation and equilibrium titrations. We illustrate this point using FPR binding to the thrombin mutant S195A, inactivated at the catalytic S195 to prevent hydrolysis (30). Two relaxations were reported for this case, along with equilibrium titrations (Fig. 3, *A* and *B*). Analysis of the slow relaxation $\alpha_{2}\left(x\right)$ yields α(0) = 0.60 ± 0.06 s^−1^, α(∞) = 17 ± 2 s^−1^, $x_{m}$ = 4.3 ± 0.4 μM, and $\alpha \left(x_{m}\right)$ = 8.8 ± 0.9 s^−1^ (Fig. 3*A* and Table 2). Equilibrium binding of FPR gives a value of $K_{d,app}$ = 0.28 ± 0.03 μM (Fig. 3*B* and Table 2). Interpretation of the binding mechanism in terms of CS gives $k_{12}$ = 17 ± 2 s^−1^ and $k_{off}$ = 0.60 ± 0.06 s^−1^. Application of Equation 11 yields $k_{on}$ = 2.4 ± 0.2 μM^−1^s^−1^ and Equation 9 gives $\hat{\alpha }\left(0\right)={k_{12}+k}_{21}$ = 19 ± 2 s^−1^ from which a value of $k_{21}$ = 2.0 ± 0.2 s^−1^ can be obtained (Table 2). These values are in excellent agreement with the ones reported originally from analysis of the two independent relaxations (Table 2) (30). When used in Equation 3 to predict the dependence of the fast relaxation $\alpha_{1}\left(x\right)$ on [FPR], the curve (discontinuous line) deviates little from experimental data (Fig. 3*A*, top portion of the panel). Values derived from analysis of the two relaxations are obviously more rigorous and should be preferred when available, but the accuracy of values obtained by application of the method based on Equation 11 is noteworthy. The discrepancies in Table 2 between values of $k_{21}$ for CS or $k_{off}$ and $k_{32}$ for IF are small and the fast relaxation ($\alpha_{1}$ in Fig. 3*A*) predicted from knowledge of a single, saturable relaxation ($\alpha_{2}$ in Fig. 3*A*), and equilibrium binding measurements (Fig. 3*B*) is in excellent agreement with experimental data.

### The method as a litmus test for IF and CS

The method also offers a simple test of whether the experimental data under consideration require more complex binding mechanisms than IF or CS. This is an important property of the method that may prompt reconsideration of current interpretations of ligand binding in terms of IF or CS made from analysis of a single saturable relaxation. The denominator of Equation 11 introduces a constraint on the relative values of $K_{d,app}$ and $x_{m}$. If the resulting term is negative, the model independent parameters (Equations (5), (6), (7) and 10) of the slow relaxation cannot be interpreted in terms of IF or CS. The data describing the kinetic relaxation (Fig. 1, *A* and *B*) and equilibrium binding (Fig. 1*C*) become incompatible when interpreted with Equations 9 and 10 and more elaborate mechanisms should be considered. We illustrate this point for the case of FPR binding to prothrombin, which is the physiological zymogen precursor of thrombin (23). Prothrombin is composed of a protease domain identical to prethrombin-2 linked to additional kringles and Gla domains that are shed during activation to thrombin by prothrombinase in the penultimate step of the coagulation cascade (23, 31). Because prothrombin and prethrombin-2 share the same protease domain, binding of FPR may be expected to take place with a similar affinity and mechanism in the two zymogens. Indeed, binding of FPR gives rise to a single saturable relaxation in the two proteins (Fig. 2*A*) but the relaxation increases with [FPR] for prethrombin-2 and decreases for prothrombin (30). Although this rules out IF as a mechanism for FPR binding to prothrombin, it does not rule out the possibility that prethrombin-2 and prothrombin both obey CS with similar values of $k_{off}$ and different values of $k_{12}$ (1, 30). However, this recent interpretation is now called into question by the method based on Equation 11. The saturable relaxation for prothrombin shows α(0) = 45 ± 4 s^−1^, α(∞) = 6.9 ± 0.7 s^−1^, a midpoint of the transition $x_{m}$ = 54 ± 5 μM and $\alpha \left(x_{m}\right)$ = 26 ± 3 s^−1^ (Fig. 2*A*). Measurements of the equilibrium binding curve for FPR, under identical experimental conditions, give a value of $K_{d,app}$ = 195 ± 20 μM (Fig. 2*B*) and application of Equation 11 returns a negative estimate for $k_{on}$ (Table 2). Therefore, FPR binding to prothrombin is neither consistent with IF (Equation 1) nor CS (Equation 2) and requires inclusion of additional steps in the kinetic mechanism that either merge IF and CS or include multiple conformations capable of interacting with ligand at the active site, as discussed elsewhere (1).

## Discussion

The method presented in this study can be used to resolve the four independent kinetic parameters of a two-step mechanism of ligand binding like IF or CS when only the slow, saturable relaxation from rapid kinetics is accessible to experimental measurements (Fig. 1, *A* and *B*). This is a common situation encountered in practice and limits resolution of the four independent rate constants of the IF and CS mechanisms. The limitation is particularly problematic for establishing the value of $k_{on}$, which is derived from the slope of the fast relaxation in the limit of high ligand concentrations (1). The value of $k_{on}$ is limited by diffusion to about 6.5 × 10^8^ M^−1^s^−1^ under physiological conditions (32, 33) and its biological relevance stems from the evolutionary optimization achieved to modulate the rate of productive encounter between the ligand and its target. Values of $k_{on}$ near the diffusion limit are observed for toxins that neutralize the function of ion channels (34) or neural transmission (35) to cause paralysis in the prey, inhibitors of coagulation factors that cause the prey to bleed to death (36), and molecules that neutralize the action of nucleases in degrading RNA (33, 37). In addition, optimization of drug design usually starts with $k_{on}$, followed by increase of the so-called “residence time” through modulation of $k_{off}$ (5). Hence, a method that can resolve $k_{on}$ from analysis of experimental data bears general practical relevance and advances basic knowledge.

Application of the method to the analysis of ligand binding to prethrombin-2, the immediate zymogen precursor of thrombin, reveals new details on the zymogen to protease conversion in the trypsin family of proteases (24, 25, 26). A long-held view in the field is that the zymogen features an “immature” active site that precludes efficient ligand binding. The defect is corrected by organization of the active site and primary specificity pocket through the Huber-Bode mechanism of zymogen activation (27). However, recent evidence shows that inhibitors do bind to the active site of zymogens like prethrombin-2 and prothrombin (28). Structural studies by X-ray crystallography (22, 38, 39, 40, 41), ^19^F NMR (42), and cryo-EM (31) show that the architecture of the active site of these zymogens does not differ significantly from that of the mature protease thrombin. These observations are consistent with a value of $k_{on}$ for FPR binding being similar between prethrombin-2 and thrombin (Table 2). The >100-fold difference in equilibrium binding affinity between prethrombin-2 and thrombin (Table 2) is due to the value of $k_{off}$, much slower for the mature protease. The conclusion is independent of whether binding is interpreted with IF or CS. In the context of CS, the ratio of inactive E∗ to active E forms in Equation 2, $k_{21}/k_{12}$, changes from 5:1 in prethrombin-2 to 1:4 in thrombin (Table 2). Hence, at least for the prethrombin-2 to thrombin conversion, the zymogen to protease transition has no significant effect on the rate at which the ligand binds to the active site but decreases the $k_{off}$ and shifts the preexisting *E∗*:*E* equilibrium in favor of the active E form. Future studies will establish if this conclusion applies more generally to other proteases of the trypsin-family.

The method provides values of the rate constants for IF or CS that are consistent with the independent analysis of multiple relaxations (Fig. 3*A*). This validation lends confidence to the approach when only a saturable relaxation is available to the experimentalist. Another noteworthy feature of the method is that it enables a simple assessment of whether the data obey a two-step mechanism of binding like IF and CS or require more complex interpretations. When application of Equation 11 returns a negative estimate for $k_{on}$, IF (Equation 1) and CS (Equation 2) no longer apply and more elaborate kinetic schemes (1, 18, 43) should be considered. Remarkably, this conclusion can be reached from analysis of a single, saturable relaxation and related equilibrium measurements. In the context of prothrombin activation, it offers valuable new insights on how auxiliary kringles and Gla domains affect allosterically the binding properties of the active site in the protease domain.

## Experimental procedures

The data analyzed in this study, that is, FPR binding to prethrombin-2, prothrombin, and thrombin (Figs. 2, *A* and *B* and 3, *A* and *B*) were taken from (30) without modification. The methodology used to produce the recombinant proteins, measure FPR binding by stopped-flow or equilibrium titrations is reported in (30) and references therein. Values of the slow relaxation α(x) (Fig. 1, *A* and *B*, 2*A* and 3*A*) are measured by stopped-slow as a function of the ligand concentration and derived from fit of the time evolution of the interaction according to the expression (1)

(12) $$A\left(x,t\right)=A_{0}\left(x\right)+A_{1}\left(x\right)\exp\left\{-\alpha \left(x\right)t\right\}$$

The value of $K_{d,app}$ is obtained from titration of intrinsic fluorescence at equilibrium (Figs. 2*B* and 3*B*) according to the expression

(13) $$F\left(x\right)=\frac{F_{0}K_{d,app}+F_{1}x}{K_{d,app}+x}=F_{0}\left(1-\vartheta \right)+F_{1}\vartheta$$

where $\vartheta =x/\left(K_{d,app}+x\right)$ is the fractional saturation (Figs. 1*C*, 2*B* and 3*B*). Alternatively, the value of $K_{d,app}$ can be obtained from the time course of the interaction in Equation 12 (44). At each value of the ligand concentration, the change in absorbance from the initial (t=0) to final (t=∞) state gives the equilibrium reading equivalent to F(x) in Equation 13.

## Data availability

Recombinant reagents and data presented in this study are available from the corresponding author upon reasonable request.

## Conflict of interest

The author declares that he has no conflict of interests with the contents of this article.
